# The Variation of Electrical Pulse Duration Elicits Reliable Network-Mediated Responses of Retinal Ganglion Cells in Normal, Not in Degenerate Primate Retinas

**DOI:** 10.3390/bioengineering10101135

**Published:** 2023-09-27

**Authors:** Seongkwang Cha, Jungryul Ahn, Seong-Woo Kim, Kwang-Eon Choi, Yongseok Yoo, Heejong Eom, Donggwan Shin, Yong Sook Goo

**Affiliations:** 1 Department of Physiology, College of Medicine, Chungbuk National University, Cheongju 28644, Republic of Korea; chaseongkwang@gmail.com (S.C.); vjahn@hanmail.net (J.A.); 2 Horang-I Eye Center, Seoul 07999, Republic of Korea; vitreokim@gmail.com; 3 Department of Ophthalmology, College of Medicine, Korea University, Seoul 08308, Republic of Korea; kwangu2@hanmail.net; 4 School of Computer Science and Engineering, Soongsil University, Seoul 06978, Republic of Korea; yyoo@ssu.ac.kr; 5 Laboratory Animal Center, Osong Medical Innovation Foundation, Cheongju 28160, Republic of Korea; eomheejong@kbiohealth.kr (H.E.); violetaromas@naver.com (D.S.)

**Keywords:** N-methyl-N-nitrosourea (MNU), retinal degeneration (RD), primate RP model, retinal prosthesis, multi-electrode array (MEA)

## Abstract

This study aims to investigate the efficacy of electrical stimulation by comparing network-mediated RGC responses in normal and degenerate retinas using a N-methyl-N-nitrosourea (MNU)-induced non-human primate (NHPs) retinitis pigmentosa (RP) model. Adult cynomolgus monkeys were used for normal and outer retinal degeneration (RD) induced by MNU. The network-mediated RGC responses were recorded from the peripheral retina mounted on an 8 × 8 multielectrode array (MEA). The amplitude and duration of biphasic current pulses were modulated from 1 to 50 μA and 500 to 4000 μs, respectively. The threshold charge density for eliciting a network-mediated RGC response was higher in the RD monkeys than in the normal monkeys (1.47 ± 0.13 mC/cm^2^ vs. 1.06 ± 0.09 mC/cm^2^, *p* < 0.05) at a 500 μs pulse duration. The monkeys required a higher charge density than rodents among the RD models (monkeys; 1.47 ± 0.13 mC/cm^2^, mouse; 1.04 ± 0.09 mC/cm^2^, and rat; 1.16 ± 0.16 mC/cm^2^, *p* < 0.01). Increasing the pulse amplitude and pulse duration elicited more RGC spikes in the normal primate retinas. However, only pulse amplitude variation elicited more RGC spikes in degenerate primate retinas. Therefore, the pulse strategy for primate RD retinas should be optimized, eventually contributing to retinal prosthetics. Given that RD NHP RGCs are not sensitive to pulse duration, using shorter pulses may potentially be a more charge-effective approach for retinal prosthetics.

## 1. Introduction

Despite the growing understanding of retinitis pigmentosa (RP) pathology and advancements in electrical stimulation techniques, current retinal prostheses exhibit limited efficacy and insufficient spatial resolution. RP patients lose their vision due to progressive retinal degeneration (RD) initiated by a loss of photoreceptors [1]. While the photoreceptor disappears, the inner retina is preserved until the end stage of the RD [2]. Retinal prostheses aim to restore their vision by evoking the spikes of surviving retinal ganglion cells (RGCs) with electrical stimulation instead of a light stimulus [3,4]. However, even with the high-density electrode array of the retinal prostheses, they show limited performance, inducing poor visual acuity (less than 20/420) in device-implanted patients [5,6]. This result suggests that the improvement in the spatial resolution of artificial vision is limited through a hardware approach.

Therefore, understanding the physiological and anatomical properties of the retinal network of degenerate retinal neurons is crucial for advancing retinal prostheses. Although the morphology of RGCs is preserved throughout the degeneration process until the end stage of the RD, the RD leads to changes in the physiological and anatomical features of the retinal network compared with normal retinas [7]. Through the progressive disappearance of the photoreceptor, the remaining retinal neurons migrate and form an abnormal synaptic connection [7]. The degenerate retina induces changes in the electrophysiological properties of RGCs, such as the appearance of an abnormal oscillation of the local field potential [8], the correlated firing of spikes among RGCs [9], multiple responses to a single pulse of electrical stimulation [10], and a reduced consistency in electrically evoked network-mediated responses [11]. Thus, an understanding of the RD network is a prerequisite for developing a retinal prosthesis for the blind.

Animal models for RD have been identified across various animal species, such as mice, rats, rabbits, cats, dogs, sheep, pigs, and non-human primates (NHPs) [12,13]. The retinal anatomy of NHPs, especially the eye size and structure of the fovea, is more similar to that of humans than other animal models [14,15,16]. Recently, only a few NHP RD models have been identified using gene editing of PDE6C and BBS7 [13]. However, these genetic RD models do not fully mimic human RP because they show macular, not peripheral, degeneration [17,18]. Our previous study [19] established an NHP RP model with the cynomolgus monkey using N-methyl-N-nitrosourea (MNU), which induces rod-specific retinal degeneration.

Epiretinal prosthesis induces artificial vision by eliciting RGC spikes directly. The advantage of epiretinal stimulation is that RGC spikes are evoked within 10 ms as a 1:1 stimulus-locked pattern [20,21,22]. However, the disadvantage of epiretinal stimulation is that RGC soma and passing axons are stimulated, hindering the spatial resolution of the prosthesis [22,23,24]. Although a soma-specific stimulation protocol is being developed, the success rate is less than 30% [25].

The subretinal prosthesis stimulates bipolar cells (BCs), then, via synaptic relay, RGC fires spike, known as a “network-mediated” response. The advantage of subretinal stimulation is it generates bursts of spikes [26], resembling the ON retinal response to a short pulse of light. Because the retinal network is used as incident light produces physiological signaling, an indirect, network-mediated activation has long been thought to be more advantageous than a direct activation in mimicking natural spiking patterns [6]. However, the retinal reorganization associated with degeneration will likely alter the normal network responses to subretinal stimulation [7], potentially limiting the benefits of engaging the retinal circuitry. For example, the correlated activity due to retinal degeneration inhibits focal response to electrical stimulation [9]. Retinal degeneration also reduces the consistency of network-mediated RGC responses [11].

However, to the best of the authors’ knowledge, any characteristics of the network-mediated RGC response with electrical stimulation are rarely investigated in the primate model. Therefore, we used normal and MNU-induced RD monkeys to compare network-mediated RGC responses in normal and degenerate RD retinas. We also investigated the efficacy of electrical stimulation in RD monkeys by varying pulse amplitude and pulse duration and its implications for understanding the degenerate retina of human RP patients.

## 2. Materials and Methods

### 2.1. Animals

This study used adult male cynomolgus monkeys (*Macaca fascicularis*) with normal (*n* = 3) and outer RD induced by MNU (*n* = 3) [19]. The monkeys had a mean age of 53.6 ± 2.2 months and a mean body weight of 3.7 ± 0.4 kg. All procedures were performed in compliance with the ARRIVE guideline. This study was approved by the Institutional Animal Care and Use Committee of Osong Medical Innovation Foundation, Cheongju, Republic of Korea (KBIO-IACUC-2020-054-4). The subjects were sacrificed approximately 14–21 weeks after MNU administration. The housing condition, how to euthanize, and the detailed methods of the operation for vitrectomy and injection of MNU were described in our previous study [19].

### 2.2. Infrared (IR) and Spectral-Domain Optical Coherence Tomography (OCT) Image Acquisition and Analysis

An OCT image of each monkey was taken twice before the MNU injection and before the sacrifice using a Spectralis OCT machine (Heidelberg Engineering GmbH, Heidelberg, Germany). The OCT image was acquired in the 55° range with the macular region at the center of the image. The image acquisition and analysis were described in our previous study [19]. The area of the macaque monkey’s fovea and macula were determined to be 0 to 0.6 mm per degree and 0.6 to 2 mm per degree, respectively [14]. Three nuclear layers in the retina, the ganglion cell layer (GCL), the inner nuclear layer (INL), and the outer nuclear layer (ONL), were identified by three dark layers on the OCT image.

### 2.3. Retinal Preparation and In Vitro Recording

**Figure 1:**
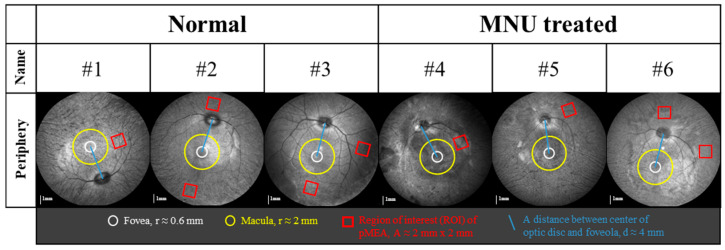
The location of the retinal patch for MEA recording. Near-IR fundus autofluorescence (FAF) images in experimental monkey eyes were taken during OCT imaging.

**Figure 2:**
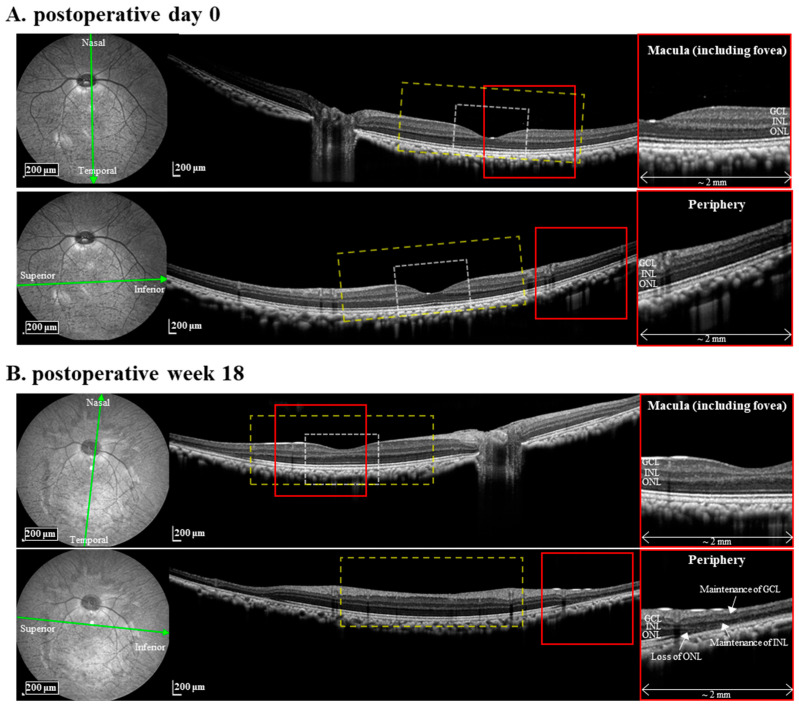
MNU induces rod photoreceptor-specific retinal degeneration in the monkey retina. The white and yellow box in the OCT image represents an area of the fovea and the macula, respectively. The zoomed-in region of the red box is shown in the right panel.

The enucleation of the eye was performed under general anesthesia, which involved intramuscular administration of 0.04 mg/kg of atropine (JEIL PHARMACEUTICAL. Co., Ltd., Seoul, Republic of Korea), 15 mg/kg of ketamine (YUHAN, Seoul, Republic of Korea), and 60 μg/kg of domitor (Orion Pharma, Espoo, Finland). After the enucleation of the eyeball, using a dissecting microscope (SZX7, Olympus, Tokyo, Japan), the eye cup, with the anterior segments removed (cornea, pupil, lens, vitreous, etc.), was observed to identify the structure of blood vessels and the optic disc, which helped to confirm the anatomical position of the retina. Moreover, when observing the primate eye with a dissecting microscope, the concave fovea and the yellow color of the macular lutea can be observed, which allows for the rough identification of the position of the macula [27]. The retina was isolated from the sclera and retinal pigment epithelium and cut into approximately 2 mm × 2 mm patches. The retinal patches for MEA recording were obtained from the peripheral region that showed loss of the ONL (Figure 1 and Figure 2). The location of the retinal patches for MEA recording was then overlapped with the FAF image to display it, as shown in Figure 1. All retinas were prepared under near-infrared (IR) illumination in an artificial cerebrospinal fluid (ACSF) solution (124 mM NaCl, 10 mM Glucose, 1.15 mM KH_2_PO_4_, 25 mM NaHCO_3_, 1.15 mM MgSO_4_, 2.5 mM CaCl_2_, and 5 mM KCl, Sigma-Aldrich, St. Louis, MO, USA) bubbled with 95% O_2_ and 5% CO_2_ to maintain a pH of 7.3–7.4 at 25 °C. The flattened retinal patch was attached to a multielectrode array (60pMEA200/30iR-Ti, Multichannel Systems GmbH, Reutlingen, Germany) with the GCL facing down.

To record the responses of RGCs, a data acquisition system (MEA 60 system, Multichannel Systems GmbH, Reutlingen, Germany) was used. Multi-electrode recordings from the retinal patch were obtained using the MEA, which had 59 titanium nitride (TiN) active electrodes with an electrode diameter of 30 μm and inter-electrode distance of 200 μm in an 8 × 8 grid layout. We continuously perfused with the oxygenated fresh ACSF to the retinal tissue on the MEA via tiny pores in the porous polyimide foil isolator using a peristaltic perfusion system (1–3 mL/min) during recording. The retinal activities were recorded after waiting 20 min to stabilize the retinal tissue attached to the MEA. Detailed information about the MEA composition and MEA 60 system was described in our previous study [28].

The electrical stimulation consisted of symmetrical cathodic phase-1st biphasic pulses. Biphasic current pulses were applied 20 times, once per second (1 Hz), for each stimulus condition. Using a stimulus generator (STG 1004, Multichannel Systems GmbH, Reutlingen, Germany), the current pulse train was delivered to the retinal patch through one of fifty-nine channels, with the remaining channels serving as recording electrodes. Pulse durations of 500, 1000, 2000, and 4000 μs/phase and pulse amplitudes of 1, 5, 10, 20, 30, 40, and 50 μA/phase were applied.

### 2.4. Data Analysis

The raw trace of the MEA recording was processed with a 100 Hz-cut-off high-pass filter. Spike sorting software (Offline Sorter^TM^ v4, Plexon Inc., Dallas, TX, USA) processed the filtered signal. Finally, the isolated timestamp of RGC spikes was analyzed with commercial analysis software (NeuroExplorer^®^ v5, Nex Technologies, Lenora, KS, USA) and custom-made MATLAB (MathWorks, Natick, MA, USA) codes.

We defined the “network-mediated” RGC responses as those occurring 10 ms after stimulus onset to exclude either the stimulus artifact or the direct RGC response occurring within 10 ms after stimulus onset [20,22,29]. We calculated the evoked spike number to quantify the electrically evoked RGC responses. First, we determined the average spike number before (or after) stimulation by counting the spikes during the 200 ms before (or after) stimulus onset across 20 trials. The difference between the average spike number after and before stimulation represented the electrically evoked RGC response. Our research is solely focused on the network-mediated response of RGCs for two reasons. Firstly, we demonstrated that the direct response within 10 ms after stimulus onset always overlapped with the stimulus artifact in our previous paper [29]. Although we have developed an artifact subtraction algorithm, it can be time-consuming and difficult to apply. Therefore, we have opted not to consider the direct response. Secondly, our Korean retinal prosthesis team is currently working on a subretinal, not epiretinal, prosthesis.

The stimulus threshold was defined as the current amplitude at which the number of RGC spikes per pulse was 0.5, meaning that ten stimulation trials out of twenty stimulation trials evoked one RGC spike/pulse, and the success rate of the stimulation trial was 50%.

Threshold charge density was calculated using the following equation:

(1) $$Threshold\;charge\;density=\frac{\left(I\times D\right)}{\left(\pi \times r^{2}\right)}$$

where *I* is the threshold amplitude, *D* is the pulse duration, and *r* is the MEA electrode radius (15 μm). The threshold charge and threshold charge density were calculated from the 1st phase of the biphasic charge-balanced stimulus pulse.

The statistical analysis was performed using commercial software (version 24) to understand the characteristics of the obtained data and further derive correlations between variables (IBM SPSS Statistics 24, International Business Machines Co., Endicott, NY, USA). A paired Student’s *t* test was performed for statistical analysis between the two groups. The statistical analysis among the three and more groups was performed with ANOVA, and post hoc analysis was performed with Tukey’s HSD and Duncan’s tests.

## 3. Results

### 3.1. MNU Induces Rod Photoreceptor-Specific Retinal Degeneration in Monkey Retinas

Figure 1 shows an example OCT image of one of the three MNU-injected monkeys. In both the macula and the peripheral region, all retinal layers, from the retinal pigment epithelium layer to the nerve fiber layer, were maintained at postoperative (postop) day 0 (Figure 2A). At postop week 18, while the peripheral region showed a disappearance of the outer retina, including OPL, ONL, and the inner/outer segment of the photoreceptor, the macula did not change compared to postop day 0 (Figure 2B). The others also showed peripheral region-specific outer RD at postop weeks 14 and 21, respectively.

### 3.2. RGCs in RD Monkey Retinas Show Imprecise Spike Response Time with Repeated Electrical Stimulation

We recorded 451 and 309 RGCs that showed spontaneous spike firing from the three normal and three RD retinas, respectively. Among these RGCs, only 301 and 184 RGCs responded to a current pulse of 50 μA and 500 μs in the normal and RD retinas, respectively. Previously, we reported that while RGCs closer to the stimulation site were more effectively activated in the WT retina, the RD RGCs showed a wide-spreading spatial distribution of electrically evoked RGC spikes. Specifically, no change was noted in the response intensity of the RD RGCs up to 800 µm (*rd1* and *rd10* P56) or 600 µm (RD macaques) to stimuli greater than 30 µA [9]. Therefore, we selected one channel among the center channels of the MEA (e.g., channels 34, 44, and 54) as the stimulation channel to constrain the distance from the stimulation to the recording channels to within 1000 µm.

**Figure 3:**
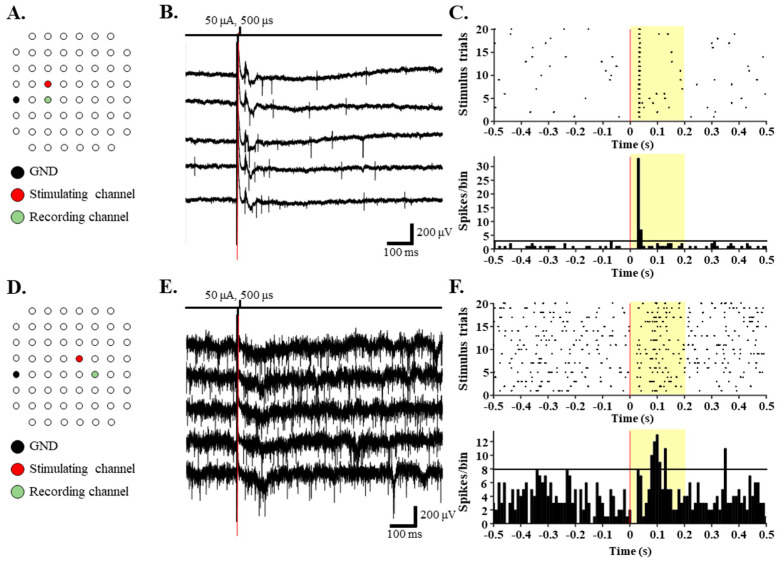
The single electrical pulse evokes a network-mediated response of RGCs within 200 ms from stimulus onset. (**A**,**D**) Locations of stimulating (red) and recording (green) electrodes that recorded the RGCs shown in B and E in the MEA recordings. Raw traces of RGCs when a current pulse of 50 μA amplitude and 500 μs duration is applied in normal (**B**) and RD (**E**) retinas. (**C**,**F**) Raster plot and post-stimulus time histogram (PSTH) from the RGCs shown in (**B**,**E**). The electrically evoked RGC response occurred in the highlighted time window (yellow area in **C**,**F**). The bin size for the PSTH is 10 ms. The horizontal lines in Figure 3C,F show 99% confidence interval, which is the guideline for meaningful PSTH peak.

Figure 3 shows an example of an RGC responding to the stimulation pulse. The RD RGCs showed a higher amplitude of baseline noise and hyperactivity of spontaneous spike firing than the normal retinas (Figure 3B,E). The RGCs in the normal retinas generated a consistent spike firing to the repeated stimulation pulse (Figure 3C). On the other hand, the RD RGCs did not precisely respond to the repeated stimulation pulse (Figure 3F).

### 3.3. RD Monkey Retinas Need Higher Threshold Charge Density Thane Normal Monkey Retinas

**Figure 4:**
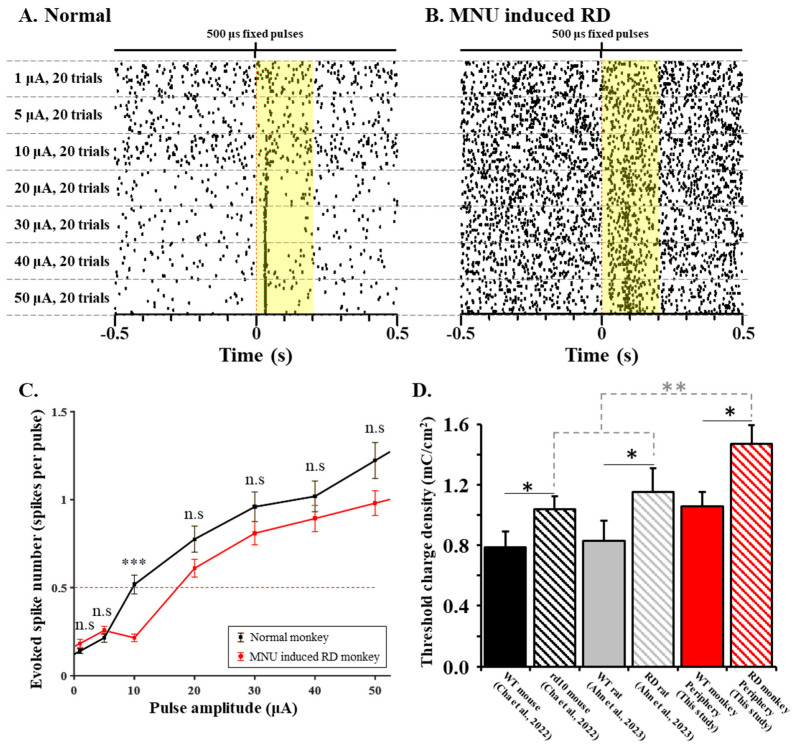
RD requires a higher threshold charge density for eliciting an RGC spike than the normal retina, regardless of animal species. (**A**,**B**) The raster plot of a representative RGC stimulated by current pulses ranging from 1 to 50 µA with a fixed pulse duration of 500 μs. The electrically evoked RGC response occurred in the highlighted time window (yellow area). (**C**) The response curve of normal and RD RGCs. There was no statistical difference between normal and RD monkeys at the same amplitude condition except at a 10 μA amplitude (***; *p* < 0.001). The “n.s” represents “not significant”. The red dashed line represents the threshold level (0.5 spikes per pulse). (**D**) The calculated threshold charge density of the normal and RD RGCs in rodents and monkeys from our previous studies [10,28] and this study (*; *p* < 0.05). The statistical difference among species was shown (**; *p* < 0.01). The error bar is the standard error of the mean (SEM).

To investigate the RGC response range to changing pulse amplitudes, we applied stimulus amplitudes ranging from 1 to 50 µA with a fixed pulse duration of 500 μs. Our previous study identified this stimulus range (from 1 to 50 µA with a fixed pulse duration of 500 μs) as the optimal parameter to induce RGC spiking in WT and RD mice [10]. In both the normal and RD monkeys, more RGC spikes were elicited with the increasing pulse amplitude. However, a spike firing with low temporal precision within 100 ms from the stimulus onset was observed in the RD monkeys (Figure 4A,B).

The primate RD model requires a higher threshold charge density for eliciting a network-mediated RGC response than the rodent RD model (RD monkey; 1.47 ± 0.13 mC/cm^2^, *rd10* mouse; 1.04 ± 0.09 mC/cm^2^, and RD rat; 1.16 ± 0.16 mC/cm^2^, *p* < 0.01). However, regardless of species, the RD retinas required a significantly higher threshold charge density than the normal (*p* < 0.05) retinas to evoke RGC responses (normal monkey; 1.06 ± 0.09 mC/cm^2^, WT mouse; 0.78 ± 0.10 mC/cm^2^, and WT rat; 0.83 ± 0.14 mC/cm^2^) (Figure 4C,D).

**Figure 5:**
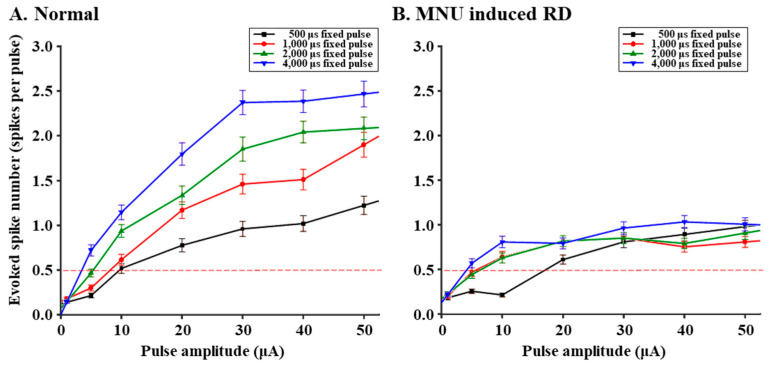
RD retina showed a smaller number of evoked spikes than normal retina when varying pulse duration. The four pulse durations (500, 1000, 2000, and 4000 μs) were applied to normal and RD monkeys. The red dashed line represents the threshold level (0.5 spikes per pulse). The error bar is the standard error of the mean (SEM).

We tested the effect of pulse durations longer than 500 μs (Figure 5). When the pulse amplitude was varied with a fixed duration of 500, 1000, 2000, and 4000 μs, the RD RGCs showed a smaller increase in the number of evoked spikes than the normal RGCs in all the stimulus protocols.

## 4. Discussion

After photoreceptor death, the RD process involves a network remodeling of the remaining inner retinal neurons [7]. Due to this retinal remodeling, the network-mediated RGC response varies with the progression of the RD [10,11]. Therefore, developing preclinical animal models that mimic most of the changes in the retinal network in human RP should be accomplished. The OCT images of our MNU-induced RD monkeys showed that their inner retinal layers are well-preserved after their outer retinal layers disappear, like in human RP (Figure 2). Although the OCT images could not show synaptic changes between the remaining neurons, several previous reports provide circumstantial evidence for synaptic changes. First, the MNU-induced RD monkeys’ outer nuclear layer disappeared when we observed them at postoperative two weeks [19]. Second, the retinal remodeling in MNU-induced RD mice occurs in their inner nuclear layers, including BCs and horizontal cells, up to 3 months after MNU injection [30]. Third, RGCs in MNU-induced RD monkeys showed an oscillation of the local field potential and hyperactivity of spontaneous firing by receiving aberrant synaptic inputs from remaining BCs at postoperative months 3 [9]. Therefore, our monkey model at postoperative weeks 14–21 is an excellent experimental model of RP patients, fully considering neural remodeling. A detailed discussion of the similarities and differences in the retina between MNU-induced RD monkeys and human RP was described in our previous study [19] based on changes in a full-field electroretinogram, OCT, FAF, and histological staining.

Our result suggests that the electrical stimulation protocol optimized in non-primate animal models may have low efficacy when adapted to primate RD since monkeys needed a higher charge density than rodents (*p* < 0.01, Figure 4D) within the RD models. Regardless of species, a higher threshold charge density is required to evoke RGC spikes in RD retinas than in normal retinas. There are two hypotheses for the higher stimulus threshold of the RD RGCs. First, gliosis caused by the hypertrophy of Müller cells and microglia is observed in rodent and NHP models induced by MNU [19,30,31]. These glial shields prevent the remaining retinal neurons from being excited via electrical stimulation. Second, the hyperactivity and oscillation of the remaining RGCs in the RD retina due to neural remodeling cause a decrease in the signal-to-noise ratio (SNR), the ratio of post-stimulus spikes to spontaneous firing [28]. As for why monkeys have a higher threshold than rodents, the more complex convergence of synaptic input in the primate retina could be an explanation. While the mouse and macaque peripheral retinas are both rod-dominant retinal networks [14], the macaque retinal network is more complex than the mouse’s. Twenty-eight types of BCs are classified in the macaque peripheral retina, which is located 3.00–3.25 mm temporal to the foveolar [32,33]. In the mouse retina, fifteen types of BCs are classified [34]. Monkey RGCs might have a more complex convergence of synaptic input from various types of BCs, including both ON- and OFF-types, than mouse RGCs. For instance, some midget ganglion cells form 161 ribbon synapses with four different BCs, making S, L, M OFF center and L+M ON surround antagonism [32]. Under electrical stimulation, all BCs might be non-specifically excited and influenced to produce RGC spikes. The more complex synaptic convergence of RGCs might lead to a higher threshold charge density in monkey RD, among many other species.

While pulse amplitudes higher than 10 μA elicit more RGC spikes, pulse durations longer than 500 μs elicit less RGC spikes in RD monkey retinas (Figure 4 and Figure 5). Conventionally, it is well-known that a long-duration pulse (over 1000 μs) more effectively induces a network-mediated RGC response due to voltage-gated Ca^2+^ channels in BCs [35]. However, our data showed that RGC spikes in RD monkeys and RD pigs [36] were not well induced when we applied a long pulse duration above 1000 μs (Figure 5). We hypothesize that the inhibitory input with GABA by electrically activated surviving horizontal cells (HCs) to BCs and RGCs limits spike firing in the RD. About 70% of HCs survive throughout the degeneration progress until the end stage of the RD after photoreceptor loss [37]. These HCs migrate toward the IPL and form abnormal synapses in the IPL [7]. Consequently, the inhibitory input to BCs from electrically activated HCs will reduce the glutamate release from BCs to RGCs. We previously reported that the shorter the pulse duration is, the more spikes of RGCs are induced under the same charge condition in the RD rats [28]. A previous report [35] shows that there is no significant difference in the RGC spike number between long-duration pulse (4000 μs, −100 μA) and short-duration pulse (less than 460 μs, ±400 μA). However, regarding the spike firing consistency in the RGCs of rd10 mice, the short-duration pulse is more effective than the long-duration pulse. Even with a lower charge of 184 nC of a short-duration pulse, the short-duration pulse elicits better results than a higher charge of 400 nC of a long-duration pulse. Since long-duration pulses were not effective in inducing RGC spikes in RD monkeys, we should consider different stimulation strategies to optimize the primate RD response. Because we have not tried a pulse duration shorter than 500 μs, it is worth trying a shorter pulse (<500 μs) in primate retinas to observe the outcome.

This study has several limitations. First, the responses of RGCs were summed, not differentiating their subtypes. However, a direct link between the RGC subtype and the electrically evoked response of the RGCs is the main interest of the artificial vision community. The ON and OFF pathways transmit two conflicting pieces of visual information about changes in light intensity to the brain in parallel [38]. The selective stimulation of RGC subtypes is expected to improve the spatial resolution of retinal prostheses by introducing center-surround antagonism. Second, this study applied a broad, not focal, electrical field to the retina, leading to electrically evoked RGC spike responses as far as 1000 μm from the stimulating electrode. Focal stimulation is expected to be more effective in improving the spatial resolution of retinal prostheses [9]. Third, the RD RGCs were not exposed to pulse durations below 500 μs either. Pulse durations longer than 500 μs were not effective in inducing RGC spikes in RD monkey retinas.

For future studies, using higher pulse amplitude modulation with short pulse durations (<500 μs) or multiple pulse stimulation with no time interval could help us optimize electrical stimulation parameters for primate RD retina. In future studies, thorough explorations of the degenerate primate retinal network will enhance the visual acuity of retinal prostheses for RP patients.

## 5. Conclusions

Regarding network-mediated RGC spikes, increasing pulse amplitudes evoke more RGC spikes in primate RD retinas, like in normal retinas. No statistical difference was found between normal and RD retinas at the same pulse amplitude condition with a pulse duration of 500 μs. More RGC spikes are evoked in normal primate retina with an increasing pulse duration from 500 to 4000 μs, but not in RD retina. Therefore, the pulse strategy for primate RD retinas should be optimized, eventually enhancing the visual acuity of RP patients implanted with retinal prosthetics. Given that RD NHP RGCs are not sensitive to pulse duration, using shorter pulses may potentially be a more charge-effective approach for retinal prosthetics.

## Data Availability

Data are contained within the article. The additional data presented in this study are available on request from the corresponding author.
